# Gene replacement therapy restores *RCBTB1* expression and cilium length in patient‐derived retinal pigment epithelium

**DOI:** 10.1111/jcmm.16911

**Published:** 2021-10-07

**Authors:** Zhiqin Huang, Dan Zhang, Shang‐Chih Chen, Luke Jennings, Livia S. Carvalho, Sue Fletcher, Fred K. Chen, Samuel McLenachan

**Affiliations:** ^1^ Centre for Ophthalmology and Visual Science The University of Western Australia Perth WA Australia; ^2^ Lions Eye Institute Nedlands WA Australia; ^3^ Centre for Molecular Medicine and Innovative Therapeutics Murdoch University Murdoch WA Australia; ^4^ Centre for Neuromuscular and Neurological Disorders The University of Western Australia Nedlands WA Australia; ^5^ Department of Ophthalmology Royal Perth Hospital Perth WA Australia; ^6^ Department of Ophthalmology Perth Children’s Hospital Nedlands WA Australia

**Keywords:** adeno‐associated virus, gene therapy, induced pluripotent stem cells, inherited retinal disease, RCBTB1, retinal pigment epithelium

## Abstract

Biallelic mutations in the *RCBTB1* gene cause retinal dystrophy. Here, we characterized the effects of *RCBTB1* gene deficiency in retinal pigment epithelial (RPE) cells derived from a patient with *RCBTB1*‐associated retinopathy and restored *RCBTB1* expression in these cells using adeno‐associated viral (AAV) vectors. Induced pluripotent stem cells derived from a patient with compound heterozygous *RCBTB1* mutations (c.170delG and c.707delA) and healthy control subjects were differentiated into RPE cells. RPE cells were treated with AAV vectors carrying a *RCBTB1* transgene. Patient‐derived RPE cells showed reduced expression of *RCBTB1*. Expression of *NFE2L2* showed a non‐significant reduction in patient RPE cells compared with controls, while expression of its target genes (*RXRA*, *IDH1* and *SLC25A25*) was significantly reduced. Trans‐epithelial electrical resistance, surface microvillus densities and primary cilium lengths were reduced in patient‐derived RPE cells, compared with controls. Treatment of patient RPE with AAV vectors significantly increased *RCBTB1*, *NFE2L2* and *RXRA* expression and cilium lengths. Our study provides the first report examining the phenotype of RPE cells derived from a patient with *RCBTB1*‐associated retinopathy. Furthermore, treatment of patient‐derived RPE with AAV‐RCBTB1 vectors corrected deficits in gene expression and RPE ultrastructure, supporting the use of gene replacement therapy for treating this inherited retinal disease.

## 1 INTRODUCTION

Mutations in the human *RCC1 and BTB domain*–*containing protein 1* (*RCBTB1*) gene have recently been associated with inherited retinal disease (IRD).^1^, ^2^, ^3^, ^4^, ^5^ Wu et al.^3^ first identified heterozygous frameshifting mutations in the *RCBTB1* gene in three cases from two unrelated Taiwanese families with Coats disease (OMIM #200216) and familial exudative vitreoretinopathy (FEVR, OMIM #133708), respectively. In contrast, the *RCBTB1*‐associated retinopathy cases reported by Coppieters et al.^1^ were all caused by biallelic missense mutations, suggesting a recessive mode of inheritance.^1^ Supportively, a recent study by Yang et al.^4^ demonstrated heterozygous truncating RCBTB1 mutations were not significantly associated with retinal disease phenotypes, while biallelic RCBTB1 mutations were associated with retinitis pigmentosa. Coppieters et al.^1^ further demonstrated reduced expression of *CUL3*, *NFE2L2* and *NFE2L2* target genes in patient peripheral blood mononuclear cells (PBMCs), suggesting that *RCBTB1* variants may impair *NFE2L2* regulation and/or ubiquitination.

To date, there have been no in vitro investigations of the effect of *RCBTB1* variants in patient‐derived retinal cells. Given the limited numbers of published studies on *RCBTB1*‐associated retinopathy, additional clinical cases and laboratory investigations of *RCBTB1*‐related ocular disease are essential for reconciling the inconsistent phenotypes and disease mechanisms reported previously. We previously identified a family with isolated IRD caused by compound heterozygous mutation in *RCBTB1* ^5^ and reprogrammed fibroblasts from the proband to produce three induced pluripotent stem cell (iPSC) lines.^2^ In the present study, we sought to utilize these patient‐derived iPSCs to produce retinal pigment epithelial (RPE) cells and determine the effects of *RCBTB1* gene deficiency in these cells. Furthermore, we report the development of AAV‐based *RCBTB1* gene therapy vectors capable of restoring *RCBTB1* expression and correcting the ultrastructural changes seen in these patient iPSC–derived RPE cells.

## 2 MATERIALS AND METHODS

### 2.1 Institutional review board approvals

Patient‐derived samples were obtained with informed consent following protocols approved by the Human Research Ethics Committee, Sir Charles Gairdner Hospital (2001‐053), Nedlands, Western Australia, Australia, and the Human Ethics Office of Research Enterprise, the University of Western Australia (RA/4/1/7916). This study was performed in accordance with the Declaration of Helsinki. The protocols used in this study were approved by the Institutional Biosafety Committee of the Harry Perkins Institute of Medical Research, University of Western Australia (NLRD 02‐2020).

### 2.2 Induced pluripotent stem cell culture

Patient iPSCs were generated as previously described.^2^ An iPSC line derived from a healthy subject (Control iPSC‐1) was obtained from a commercial provider (A18945, Thermo Fisher). Two additional clonal control iPSC lines (Control iPSC‐2 and iPSC‐3) were derived from a subject without retinal disease, using our published methods, and characterized as previously described.^2^ Generation and characterization of Control iPSC‐2 and iPSC‐3 is summarized in Figure S1. Control and patient iPSC lines were passaged using an EDTA‐based passing procedure onto Geltrex‐coated 6‐well plates in StemFlex medium (A3349401, Gibco), as previously described.^2^

### 2.3 RPE differentiation

To generate RPE from iPSCs, we made minor modifications to a recently reported RPE differentiation protocol.^6^ Briefly, iPSCs were cultured on Geltrex in 6‐well plates containing StemFlex medium. Upon reaching confluence, StemFlex medium was replaced with RPE differentiation media including DMEM/F12 (11320, Gibco) supplemented with 15% knockout serum replacement (10828028, Gibco) and 1× antibiotic‐antimycotic (15240062, Gibco) for the first 24 h. The following day, 10 mM nicotinamide (NIC, N3376‐100G, Sigma‐Aldrich) and 25 nM chetomin (C9623‐1mg, Sigma‐Aldrich) were added to the media. Media were changed daily during the process of differentiation. Chetomin was removed after two weeks of culture, while 10 mM NIC was included for two additional weeks. After 4 weeks, RPE cells displayed typical pigmented polygonal morphology and were cultured in RPE media including 70% DMEM (11995040, Gibco) and 30% DMEM/F12, supplemented with B27 (17504001, Gibco) and 1× antibiotic‐antimycotic. From Week 5, media were changed every 3–5 days. RPE passaging was performed by trypsin dissociation and expansion at Week 4 and Week 8. For experiments, RPE cells were plated into Geltrex‐coated 96‐ or 24‐well plates, Millicell Hanging Cell Culture Inserts (24‐well, 0.4 µm, Merck) or 8‐well Millicell EZ slides (Merck) at passages 3–4 and differentiated for 2–6 weeks or 6 months.

### 2.4 Quantitative PCR

RNA was harvested from RPE cells using TRIzol (Invitrogen) according to the manufacturer's instructions. RPE cDNA was synthesized using the RT^2^ First Strand Kit (Qiagen). Quantitative real‐time PCR analysis (qRT‐PCR) was conducted using the CFX Connect Real‐Time System (Bio‐Rad) with the RT^2^ SYBR Green qPCR Mastermix (Qiagen). Samples were run in triplicate, and expression levels were normalized to *GAPDH* using the ΔCT method. Significance testing was performed using the Student *t* test. Primers used are listed in Table S1.

### 2.5 Immunocytochemistry

For immunocytochemistry analyses, RPE cells were seeded onto glass coverslips in 24‐well plates or 8‐well chamber slides. Cells were fixed with 4% paraformaldehyde for 15 min and permeabilized with 0.3% Triton X‐100 in phosphate‐buffered saline (PBS) for 10 min at room temperature. The cells were then incubated in blocking buffer (5% goat serum in PBS with 0.3% Triton X‐100) for 1 h at room temperature. Primary antibodies were added and incubated at 4°C overnight, and then, slides were washed three times in PBS. Secondary antibodies were added and incubated for 2 h at room temperature. All antibodies used are listed in Table S2. Cells were imaged using the Nikon Instruments A1 Confocal Laser Microscope, and images were analysed using NIS‐Elements Viewer (version 4.11.0; Laboratory Imaging) and ImageJ 64 (ImageJ 1.44o; National Institute of Health, USA) software.

### 2.6 Scanning electron microscopy

For scanning electron microscopy (SEM), RPE cells were passaged onto Geltrex‐coated Millicell hanging cell culture inserts with RPE medium. After 4 weeks of culture, inserts were removed following Lynn's instruction^6^ and fixed in 2.5% glutaraldehyde in 0.1 M phosphate buffer (pH 7.4) for 20 min at room temperature. Critical dehydration and platinum coating were performed according to a previously published SEM sample preparation protocol.^7^ Finally, SEM samples were visualized using a field emission scanning electron microscope (SEM Zeiss 55 Supra).

### 2.7 Transepithelial electrical resistance

To assay RPE barrier function, RPE cells were seeded onto 0.33‐cm^2^ Millicell hanging cell culture inserts. At 2 weeks and 6 weeks post‐seeding, transepithelial electrical resistance (TEER) was measured using the EVOM2 voltohmmeter with the STX3 electrode set, following the manufacturer's instructions (World Precision Instruments).

### 2.8 Cilium length measurement

Primary cilia in RPE monolayers were labelled by double immunostaining for ARL13B (1711‐1‐AP, ProteinTech) and pericentrin (ab28144, Abcam). For primary cilium length measurements, maximum projection intensity images were generated from confocal stacks using NIS‐Elements Viewer (version 4.11.0; Laboratory Imaging) and cilium lengths measured using Image J64 software (National Institute of Health, Bethesda). Mean cilium lengths were compared using the Student *t* test, with *p *< 0.05 considered significant. Cilium length distributions were compared using the chi‐squared test, with *p *< 0.05 considered significant.

### 2.9 Adeno‐associated viral vector treatments

Custom AAV2/2 and AAV2/8 gene therapy vectors (referred to as AAV2 and AAV8 in this report) were manufactured by Vector Biolabs. The vectors contain the *RCBTB1* cDNA (transcript variant 1, NM_018191) with a CAG promoter and the woodchuck hepatitis virus post‐transcriptional response element. For AAV treatments, iPSC‐derived RPE cells were seeded onto 8‐well chamber slides and cultured for 6 months to obtain mature RPE monolayers. The AAV‐RCBTB1 treatments were performed using a MOI of 2 × 10^5^ vector genomes per cell, and cells were fixed for immunostaining or harvested for RNA extraction two weeks after AAV transduction.

## 3 RESULTS

### 3.1 Derivation of iPSC‐derived RPE

Retinal pigment epithelial cells were differentiated from three clonal iPSC lines derived from a patient with *RCBTB1*‐associated retinopathy,^2^ one commercially available control iPSC line (Control iPSC‐1) and two clonal iPSC lines from a healthy control subject (Control iPSC‐2 and iPSC‐3; Figure S1). RPE cells from all six iPSC lines formed ‘cobblestoned’ monolayers comprised of pigmented polygonal cells expressing the RPE markers RPE65, bestrophin 1, ZO1, MERTK, MITF, CRALBP, Na^+^/K^+^ ATPase and tyrosinase (Figure S2A‐B). Expression of *RPE65*, *MERTK*, *MITF*, *BEST1* and *PAX6* was confirmed by qRT‐PCR. Patient RPE demonstrated significantly reduced expression of *RPE65* and *BEST1*, compared with control RPE, while *MERTK*, *MITF* and *PAX6* were expressed at similar levels as control RPE cells (Figure S2C).

### 3.2 *RCBTB1* expression and *NFE2L2* activation are reduced in patient RPE

**FIGURE 1:**
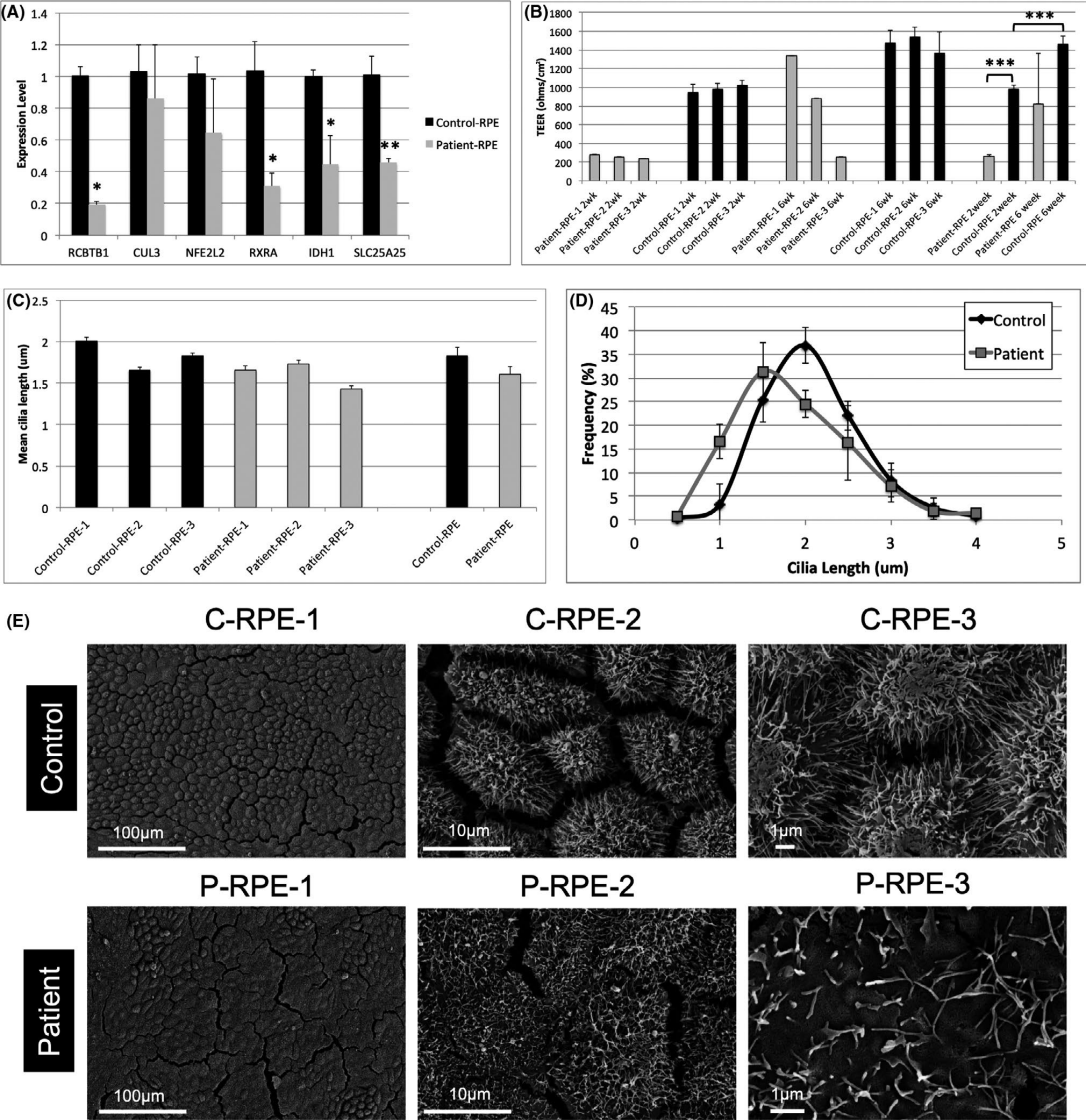
(A) Gene expression was measured by qRT‐PCR in RPE monolayers derived from three iPSC lines derived from the patient and three control lines derived from two healthy individuals, 6 weeks after seeding. Bars indicate mean *RCBTB1*, *RXRA*, *IDH1* and *SCLC25A25* gene expression values normalized to *GAPDH* and expressed as fold change compared with control levels. Error bars show standard deviation. Statistical significance was determined by the *t* test (*p < 0.05, **p < 0.01). (B) TEER was measured in RPE monolayers derived from control (*n* = 3) and patient‐derived (*n* = 3) iPSC lines at 2 weeks and 6 weeks after seeding. The bar graph shows TEER values obtained for each line, as well as the overall mean values for the control and patient RPE. Error bars indicate standard deviation. (C) Primary cilium lengths were measured in RPE cells derived from control (*n* = 3) or patient‐derived (*n* = 3) iPSC lines 6 weeks after seeding. The bar graph shows mean cilium lengths for each line, as well as the overall mean values for the control and patient RPE cells. Error bars indicate standard error of the mean. (D) RPE cells were categorized into eight groups according to primary cilium length and plotted as a frequency distribution. Each data point represents the mean frequency of RPE cells within each group, calculated from the 3 independent control or patient iPSC lines shown in C. Error bars indicate standard deviation (chi‐squared test, *p *< 0.0001). (E) SEM analysis of cultured RPE cells showing morphology in patient‐derived and control RPE cells and surface microvillus densities in patient‐derived and control RPE

*RCBTB1* expression was reduced in patient RPE compared with control RPE (*p *= 0.0002). *CUL3* expression was similar in patient and control RPE (*p *= 0.675). Patient RPE showed a non‐significant reduction in mean *NFE2L2* expression, compared with control RPE, while expression of the *NFE2L2* target genes *RXRA*, *IDH1* and *SLC25A25* was significantly reduced (*p *< 0.05; Figure 1A).

### 3.3 Development of RPE barrier function is impaired in patient RPE

Two weeks after plating, patient RPE monolayers displayed significantly reduced electrical resistance compared with control RPE monolayers (*p *< 0.001). TEER was significantly increased in 6‐week control RPE monolayers compared with 2‐week RPE monolayers (*p *< 0.001). In contrast, RPE TEER was variable in 6‐week RPE derived from the three patient lines, with one line showing TEER values similar to controls. These results suggest that although electrical resistance of RCBTB1‐deficient RPE monolayers can develop to similar levels as control RPE, the process may be delayed (Figure 1B).

### 3.4 Mean cilium length is reduced in patient RPE

Primary cilia were analysed by immunostaining for ARL13B, which labels the cilium, and pericentrin, which labels centrioles at the base of the primary cilium (Figure S2D). Mean cilium lengths ranged from 1.7 to 2.0 μm in 6‐week control RPE cells and 1.4 to 1.7 μm in patient RPE cells. Mean cilium length in patient RPE cultures was reduced across the three lines compared with the three control lines; however, the result was not significant (*p *= 0.173; Figure 1C). Analysis of cilium length distributions showed increases in the number of shorter cilia (0.5–1 μm) in patient RPE and in longer cilia (1.5–2.0 μm) in control RPE (Figure 1D). The chi‐squared analysis demonstrated patient RPE cilium length distributions differed significantly from controls (*p *< 0.001). In addition to reduced primary cilium length, ultrastructural analysis of iPSC‐derived RPE by scanning electron microscopy demonstrated the flattened appearance of patient‐derived RPE cells and reduced surface microvillus densities in patient RPE cells compared with the control cells (Figure 1E).

### 3.5 Gene therapy restores *RCBTB1* expression in patient RPE

**FIGURE 2:**
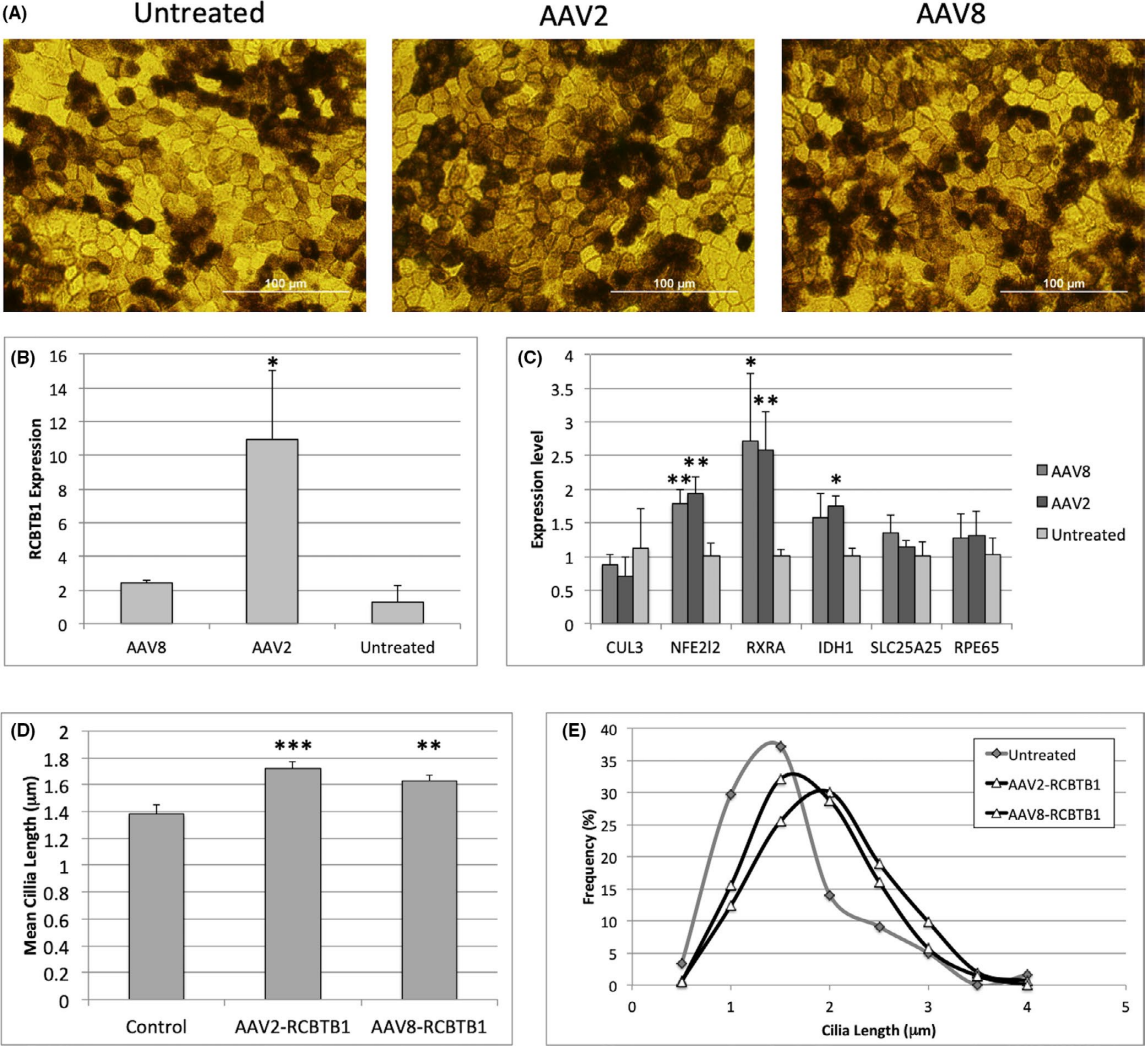
(A) Micrographs show morphology and pigmentation of mature patient‐derived RPE monolayers, six months after plating. No changes in RPE morphology were evident two weeks after treatment with AAV2 or AAV8 vectors. (B) *RCBTB1* expression was measured in patient‐derived RPE by qRT‐PCR, two weeks after treatment. Data were normalized to GAPDH expression and expressed as mean fold change compared with untreated controls (*p < 0.05). (C) Gene expression was measured by qRT‐PCR in AAV‐RCBTB1–treated and untreated patient‐derived RPE monolayers, 2 weeks after transduction. Bars indicate mean expression values normalized to control levels. Error bars show standard deviation. Statistical significance was determined by the *t* test (*p < 0.05 and **p < 0.01). (D) Mean cilium lengths in AAV2‐RCBTB1– and AAV8‐RCBTB1–treated RPE cells, compared with untreated controls. Error bars indicate standard error of the mean. Statistical significance was determined by the t test (**p < 0.01 and ***p < 0.001) (E) RPE cells were cultured, fixed and immunostained for ARL13B and pericentrin, two weeks after treatment with AAV2‐ or AAV8‐RCBTB1 gene therapy vectors. Primary cilium length distributions in AAV2‐RCBTB1– and AAV8‐RCBTB1–treated RPE are compared with untreated patient RPE (chi‐squared test, *p *< 0.0001)

To restore *RCBTB1* expression in patient‐derived RPE cells, AAV2 and AAV8 vectors carrying *RCBTB1* transgenes were designed and used to transduce mature RPE monolayers (Figure 2A). Transduction of patient RPE monolayers with AAV2‐RCBTB1 vectors resulted in significantly increased *RCBTB1* expression compared with untreated controls (11‐fold increase, *p *< 0.05), while AAV8‐RCBTB1 treatment resulted in a smaller (twofold), non‐significant increase in *RCBTB1* expression (Figure 2B). Both AAV2‐ and AAV8‐RCBTB1 treatments resulted in significantly increased expression of *NFE2L2* and its target gene and *RXRA*. Compared with untreated controls, *IDH1* expression was significantly increased in AAV2‐RCBTB1–treated patient RPE cells, but not in AAV8‐treated cells, while *SLC25A25* and *RPE65* expressions were not significantly different between treated and untreated cells (Figure 2C).

### 3.6 Gene therapy increases cilium length in patient RPE cultures

Mean primary cilium lengths were significantly increased in both AAV2‐RCBTB1– and AAV8‐RCBTB1–treated RPE monolayers (*p *< 0.001; Figure 2D). The distribution of primary cilium lengths was significantly skewed towards increased cilium lengths in AAV‐treated RPE monolayers compared with untreated controls (*p *< 0.001, chi‐squared test; Figure 2E). Together, these results demonstrate that transduction of patient‐derived RPE cells with the AAV vectors carrying *RCBTB1* transgenes restores gene expression and ciliogenesis defects associated with RCBTB1 deficiency.

## 4 DISCUSSION

In this study, we performed personalized disease modelling on a patient with *RCBTB1*‐associated retinopathy. We show that *RCBTB1* mRNA levels are markedly reduced in iPSC‐RPE derived from a patient carrying the c.170delG and c.707delA variants in the *RCBTB1* gene, which were both predicted to result in null alleles. Reduced *RCBTB1* expression in patient‐derived iPSC‐RPE was accompanied by reduced expression of oxidative stress response genes activated by *NFE2L2*, supporting previous observations made in PBMCs derived from patients with *RCBTB1*‐associated retinopathy.^1^ Additionally, we show reduced surface microvillus densities and decreased primary cilium lengths in patient RPE, compared with control RPE. Finally, we demonstrate that gene replacement therapy using AAV vectors was capable of restoring *RCBTB1* expression, leading to increased *NFE2L2* expression and cilium lengths.

Expression of the oxidative stress response gene *NFE2L2* was previously shown to be reduced in lymphocytes from patients with *RCBTB1* mutations compared with controls.^1^ In the present study, we found *NFE2L2* mRNA levels were variable between RPE monolayers derived from the three patient iPSC lines, with a non‐significant reduction in the mean expression level of this gene. Consistent with reduced *NFE2L2* expression, expression of the NFE2L2 target genes, *RXRA*, *IDH1* and *SLC25A25*, was significantly reduced compared with controls. Levels of the NFE2L2 transcription factor are tightly regulated by ubiquitination and degradation. Binding of the CUL3 adaptor protein KEAP1 to NFE2L2 induces constitutive ubiquitination and degradation of NFE2L2, keeping NFE2L2 protein levels low under basal conditions. Upon oxidative stress, conformational changes in KEAP1 cause the dissociation of CUL3, preventing degradation of KEAP1‐bound NFE2L2 protein and blocking degradation of newly synthesized NFE2L2, rapidly increasing the availability of NFE2L2. The free NFE2L2 transcription factor then binds to the promoters of target genes, activating expression of oxidative stress response proteins.^8^ Additionally, NFE2L2 binds to its own gene promoter, further increasing the NFE2L2 response through positive feedback.^9^ The reduced basal expression of *NFE2L2* and its downstream target genes in lymphocytes^1^ and RPE (Figure 1A) derived from patients with *RCBTB1* mutations suggests cells from these patients could be more susceptible to oxidative stress. However, interactions between the RCBTB1 protein and NFE2L2‐KEAP1 complexes have yet to be demonstrated and the pathogenic mechanisms underlying *RCBTB1* deficiency remain largely speculative.

In addition to changes in gene expression, we demonstrated reduced surface microvillus densities and primary cilium lengths in patient‐derived RPE cells (Figure 1C–E), indicating RCBTB1 deficiency leads to defects in RPE ultrastructure. Supportively, we found that electrical resistance was significantly reduced in two‐week RPE monolayers derived from all three patient iPSC lines, compared with controls. After 6 weeks of culture, RPE cultures differentiated from 2 of 3 patient‐derived iPSC lines showed reduced TEER compared with controls, while one patient line showed similar resistance as control RPE monolayers (Figure 1B). Reduced TEER values in 2‐week RPE monolayers suggest barrier function in RCBTB1‐deficient RPE cells may be slower to develop than in control RPE; however, since one patient line displayed TEER values similar to control levels after 6 weeks, this could be an indirect effect of RCBTB1 deficiency on cellular homeostasis during development, rather than a direct effect of RCBTB1 on tight junction formation.

Reduced primary cilium lengths in patient‐derived iPSC‐RPE cells have previously been reported in patients with retinitis pigmentosa 11 (RP11).^10^ Here, we showed primary cilium lengths were reduced in RCBTB1‐deficient RPE cells (Figure 1C–D); however, it remains unclear whether this is due to a direct involvement of RCBTB1 protein in ciliogenesis, or to indirect effects on cellular growth, differentiation or homeostasis. Extended culture of patient RPE for up to 6 months did not increase mean cilium lengths (Figure 2D), suggesting that patient RPE reached maximum cilium lengths by 6 weeks of culture. Notably, Wu et al. (2016) demonstrated that knockdown of *RCBTB1* reduced the nuclear accumulation of beta‐catenin in ARPE19 cells, suggesting *RCBTB1* deficiency may cause dysregulation of the canonical Wnt signalling pathway, which is known to play an important role in RPE differentiation. The ciliopathy and impaired barrier function phenotypes could therefore be linked to impaired RPE differentiation due to reduced canonical Wnt signalling; however, this hypothesis will require further investigation. Whether these effects are direct or indirect, cilium length provides a useful metric for evaluating treatment strategies aimed at restoring *RCBTB1* expression in RPE cells.

To restore *RCBTB1* expression in patient‐derived RPE cells, we obtained AAV vectors containing *RCBTB1* transgenes. The AAV vectors are replication‐incompetent infectious particles comprised of the outer shell of the AAV virus enclosing a functional copy of the transgene. AAV vectors have been shown to mediate efficient gene transfer to retinal cells in cell culture and animal models.^11^, ^12^ In a recently published Phase III clinical trial, injection of AAV2 vectors containing a functional copy of the *RPE65* gene into the retinas of LCA patients with biallelic *RPE65* mutations resulted in partial restoration of scotopic visual function.^13^ These exciting results have led to the approval and commercialization of the first gene therapy treatment for inherited vision loss, Luxturna^®^, demonstrating the feasibility of gene replacement therapies for IRD patients with loss‐of‐function mutations. Here, we compared the transduction efficiencies of AAV2 and AAV8 gene therapy vectors for the delivery of *RCBTB1* cDNA to patient‐derived RPE cells. Similar to previous studies,^14^, ^15^, ^16^ we found AAV2 was a more efficient transducer of iPSC‐RPE cells in vitro, with increased expression of *RCBTB1* in AAV2‐treated RPE monolayers compared with AAV8‐treated cells. However, AAV8 vectors have been reported to transduce RPE cells at higher efficiencies than AAV2 vectors in vivo,^17^ suggesting transduction efficiencies may be strongly influenced by the retinal microenvironment. Transduction of patient iPSC‐RPE with either AAV2‐RCBTB1 or AAV8‐RCBTB1 gene therapy vectors significantly increased *NFE2L2* expression and mean cilium lengths in transduced monolayers, compared with untreated iPSC‐RPE. Together, our results demonstrate that the defects in gene expression and primary cilium length present in RCBTB1‐deficient iPSC‐RPE can be corrected by AAV‐mediated delivery of *RCBTB1* cDNA.

In summary, our work demonstrates the feasibility of establishing disease‐specific, iPSC‐based systems for modelling rare human diseases and screening potential treatments. We provide the first report examining the effects of *RCBTB1* deficiency in patient‐derived RPE cells and further demonstrate the efficacy of AAV‐mediated gene therapy for restoring *RCBTB1* gene expression and correcting ciliogenesis defects in these cells. Considering the currently restricted knowledge of *RCBTB1*‐associated retinopathy, additional studies are required to elucidate the pathogenic mechanisms of *RCBTB1* deficiency.

## CONFLICT OF INTEREST

The authors have no conflicts of interest to disclose.

## AUTHOR CONTRIBUTION

**Zhiqin Huang:** Conceptualization (equal); data curation (equal); formal analysis (equal); investigation (equal); methodology (equal); project administration (supporting); writing—original draft (equal); writing—review and editing (equal). **Dan Zhang:** Methodology (equal); project administration (equal); supervision (supporting); writing—review and editing (equal). **Shang‐Chih Chen:** Investigation (equal); methodology (equal); supervision (supporting); writing—review and editing (supporting). **Luke Jennings:** Investigation (equal); methodology (equal); validation (supporting); writing—review and editing (supporting). **Livia S Carvalho:** Conceptualization (supporting); methodology (supporting); validation (equal); writing—review and editing (equal). **Sue Fletcher:** Funding acquisition (supporting); supervision (supporting); writing—review and editing (equal). **Fred K Chen:** Conceptualization (equal); formal analysis (equal); funding acquisition (lead); project administration (lead); resources (lead); supervision (lead); writing–review and editing (equal). **Samuel McLenachan:** Conceptualization (lead); data curation (equal); formal analysis (equal); funding acquisition (equal); investigation (lead); methodology (equal); project administration (lead); resources (equal); supervision (equal); validation (equal); visualization (equal); writing‐original draft (equal); writing—review and editing (equal).

## Supporting information

Figure S1‐S2

Table S1

Table S2

## Data Availability

The data that support the findings of this study are available from the corresponding authors upon reasonable request.
