# Supplementary material for: Gene replacement therapy restores RCBTB1 expression and cilium length in patient‐derived retinal pigment epithelium
Source: J Cell Mol Med. 2021 Oct 7;25(21):10020–7. doi: 10.1111/jcmm.16911 (PMC8572767; doi:10.1111/jcmm.16911)
Supplement: Supplementary file 1 — Figure S1‐S2 [file JCMM-25-10020-s003.pdf]

Gene replacement therapy restores RCBTB1 expression and cilia length in patient-derived retinal pigment epithelium

Supplementary Materials

Zhiqin Huang, Dan Zhang, Shang-Chih Chen, Luke Jennings, Livia S. Carvalho, Sue Fletcher, Fred K. Chen, Samuel McLenachan

Supplementary Figure S1: Characterization of control iPSC Lines

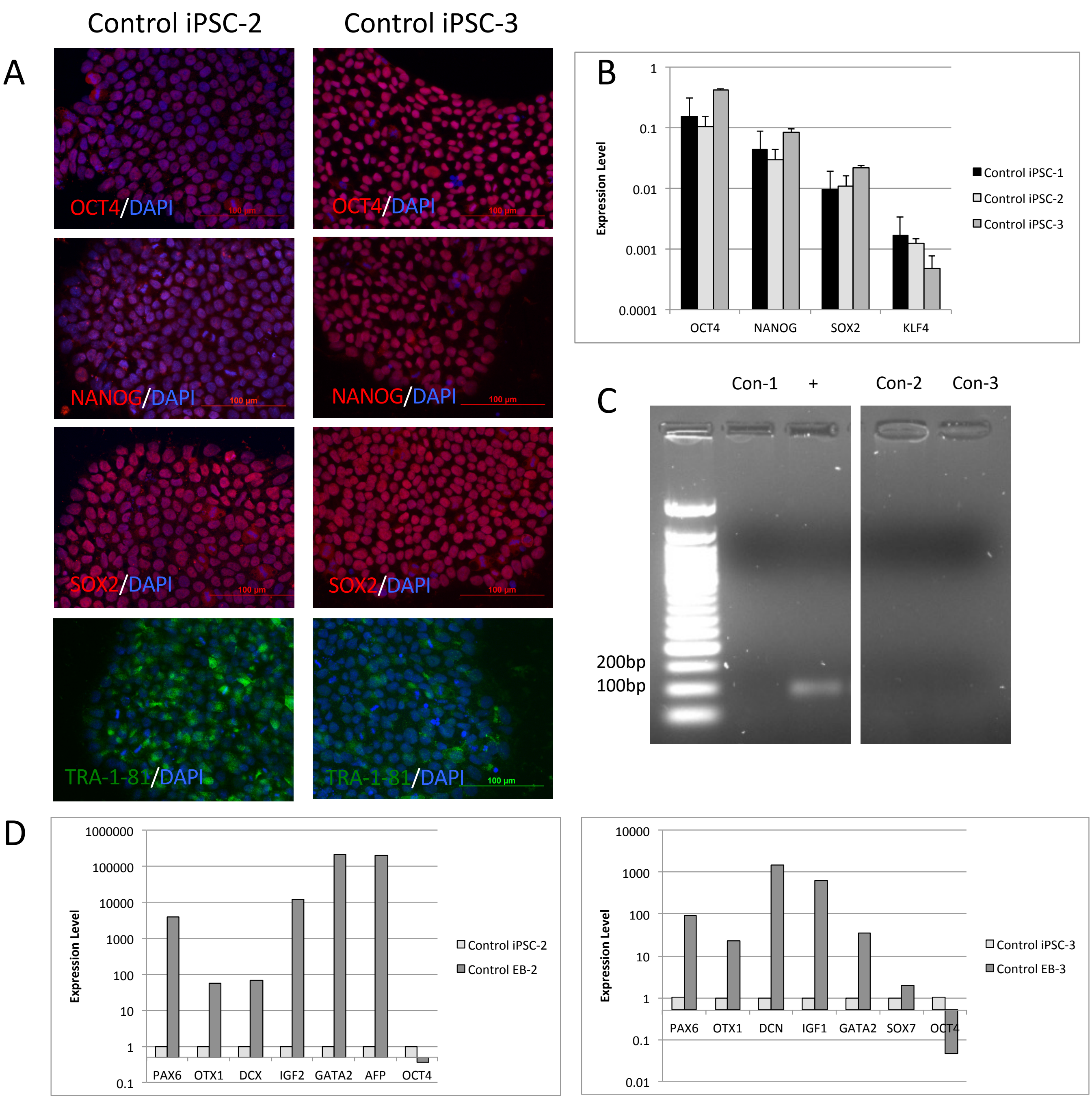

Supplementary Figure S1: Characterization of Control iPSC Lines. Dermal fibroblasts were cultured from a skin biopsy from a 24 year old male control subject. Clinical examination of the patient revealed no signs of retinal disease. Fibroblasts were reprogrammed into iPSC using the Epsiomal iPSC Reprogramming kit (Systems Biosciences) according to our previously published method (2). Briefly, fibroblasts were transfected using the NEON electroporator (ThermoFisher) and resulting iPSC colonies picked three weeks of culture. Two clonal colonies (Control iPSC-2 and -3) were established. **A**) Control iPSC-2 and -3 were shown to express the pluripotency markers OCT4, NANOG, SOX2 and TRA-1-81 by immunostaining. Merged images show expression of each marker (red or green) with nuclei labeled with DAPI (blue). Scale bars indicate 100µm. **B**) Control iPSC-1 (ThermoFisher, A18945), -2 and -3 were shown to express the pluripotency markers *OCT4*, *NANOG*, *SOX2* and *KLF4* by qRT-PCR. **C**) The absence of reprogramming episomes in Control iPSC-1, -2 and -3 was confirmed by PCR assay, as previously described (2). A 95bp amplicon was detected in a positive control sample derived from episome transfected fibroblasts (+), but not in the Control iPSC-1 (passage 40), -2 (passage 10) or -3 lines (passage 10). **D**) Control iPSC-2 (left panel) and -3 (right panel) were cultured as embryoid bodies, as previously described (2). After two weeks of spontaneously differentiation, both lines demonstrated upregulation of markers of ectoderm (*PAX6*, *OTX1*), mesoderm (*DCX*, *IGF2*, *GATA2*) and endoderm (*AFP*, *SOX7*) differentiation, as well as downregulation of *OCT4*.

Supplementary Figure S2A: Immunostaining of control iPSC-RPE

Control RPE-1

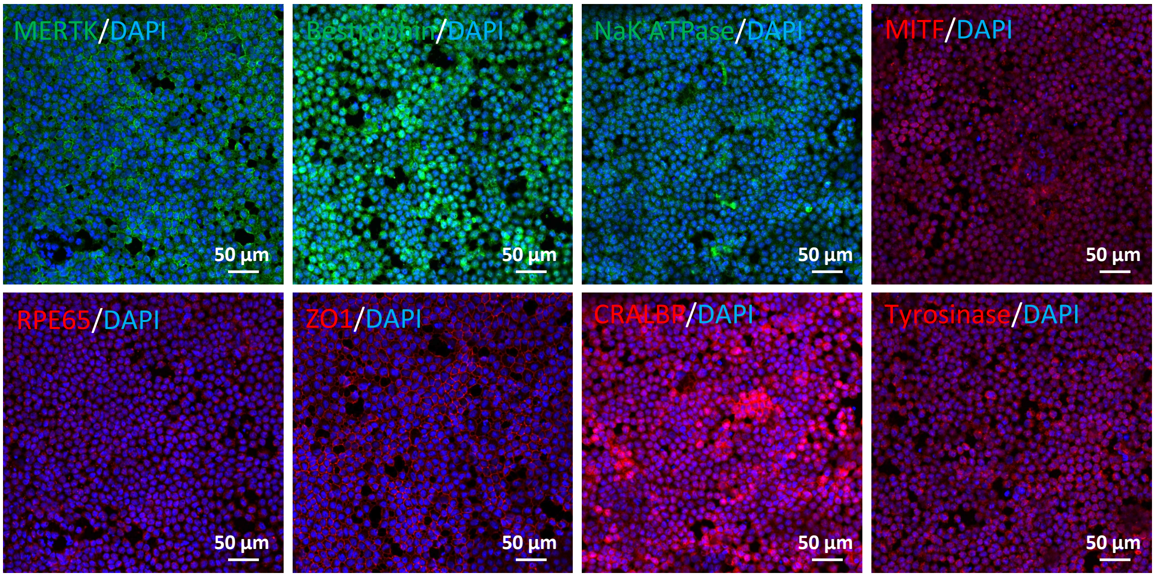

Control RPE-2

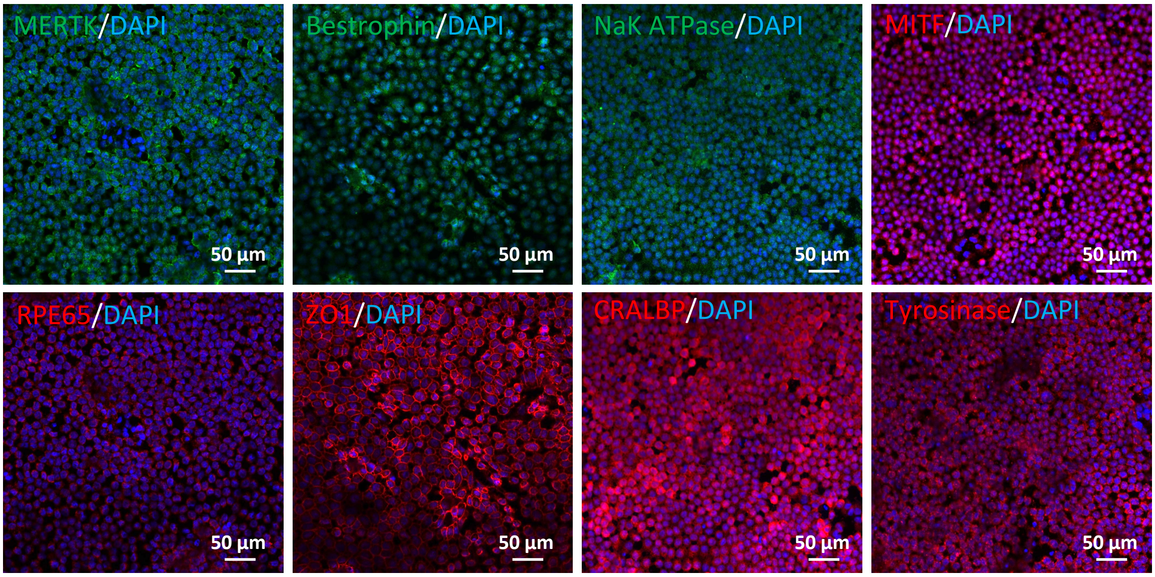

Control RPE-3

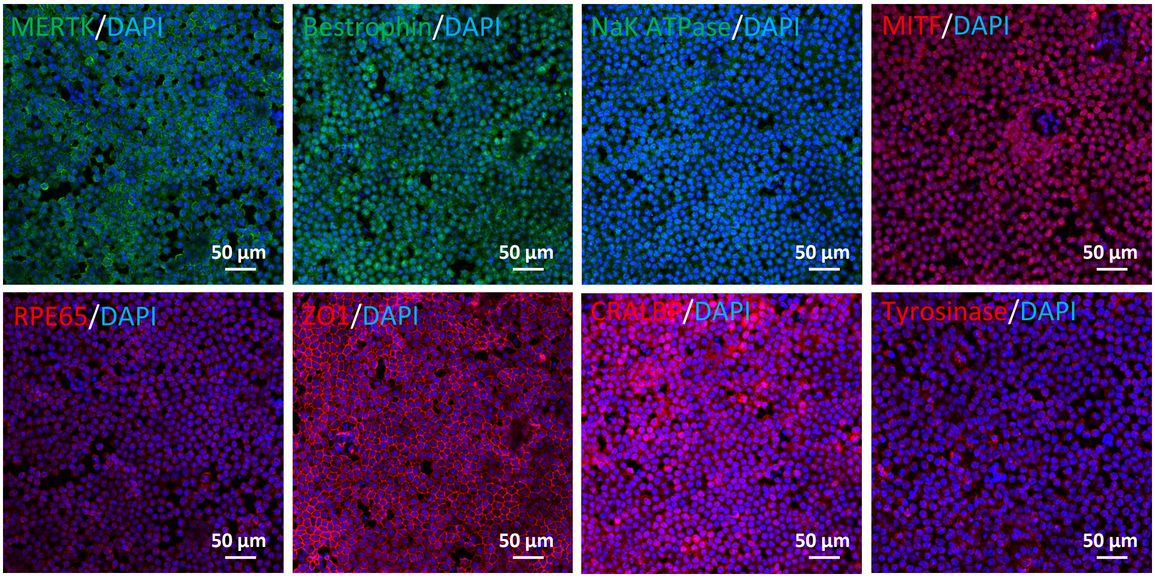

Supplementary Figure S2B: Immunostaining of patient iPSC-RPE

Patient RPE-1

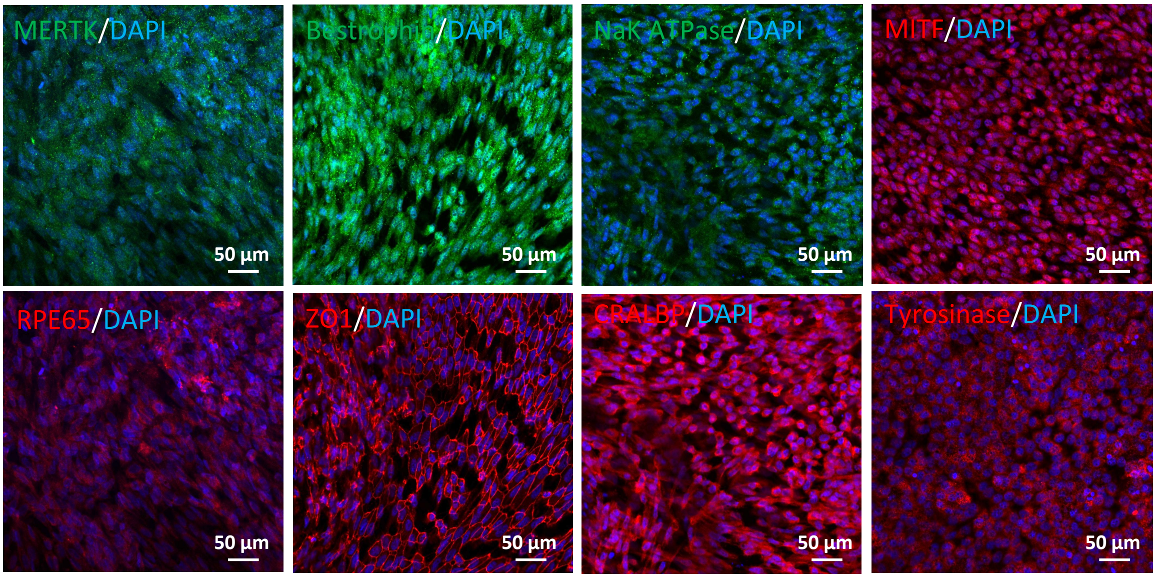

Patient RPE-2

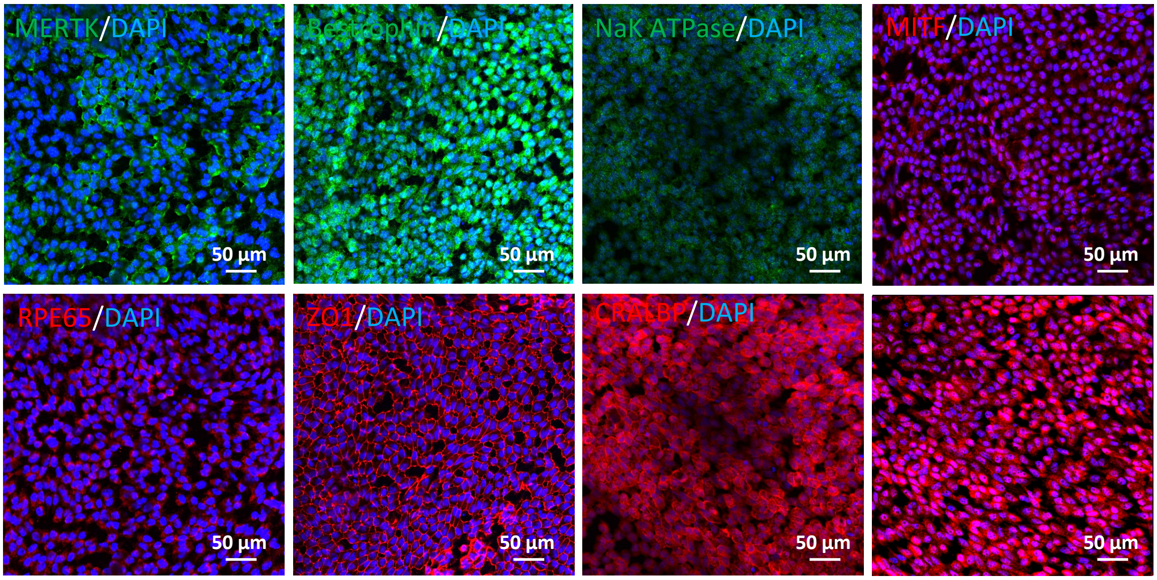

Patient RPE-3

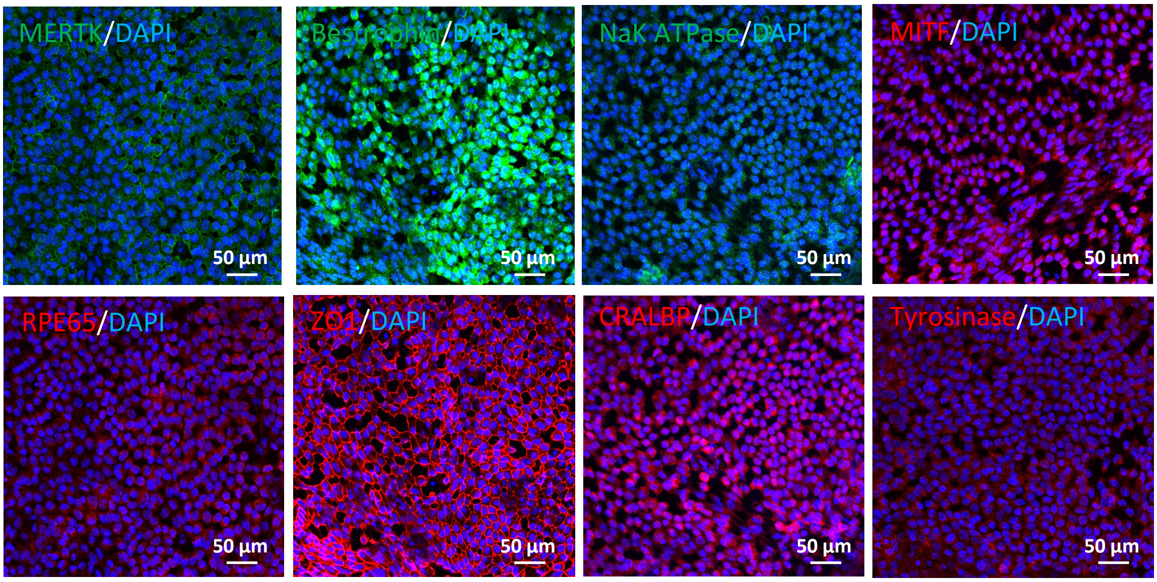

Supplementary Figure S2C: Characterization of RPE by qRT-PCR

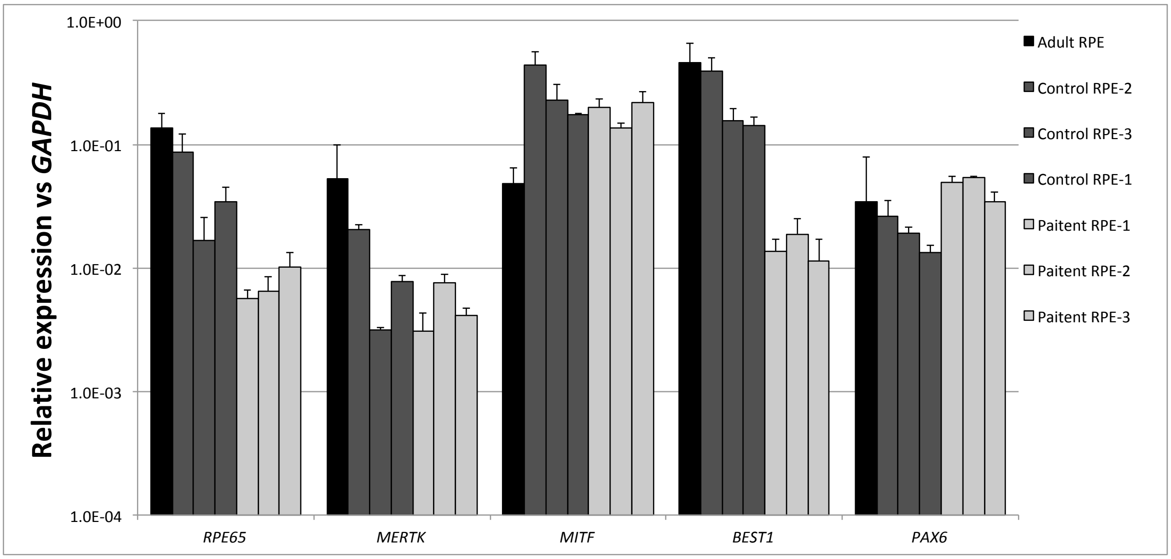

Supplementary Figure S2D: Primary cilia immunostaining

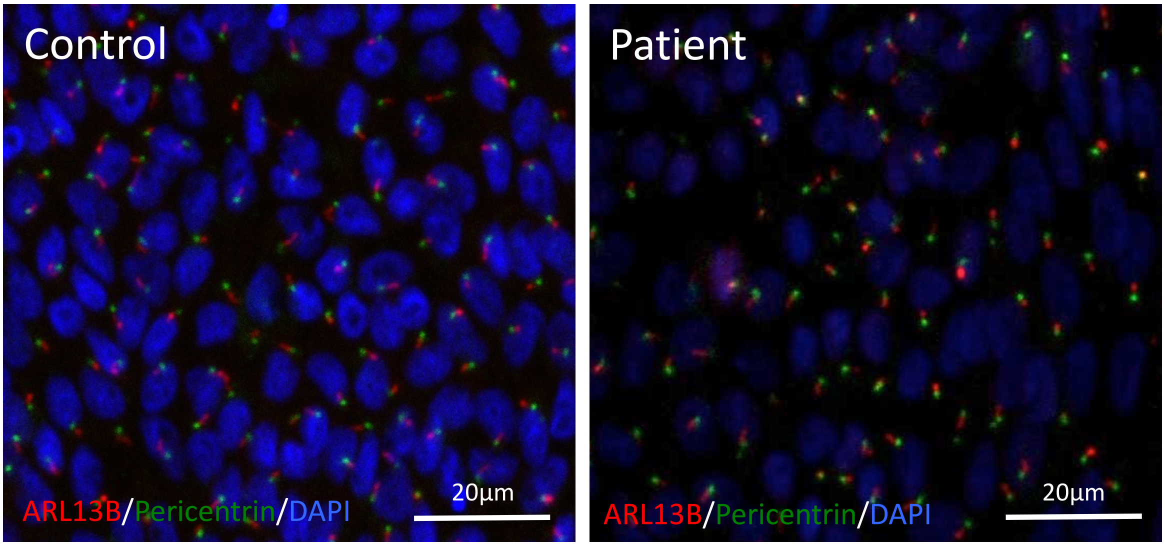

Supplementary Figure S2: Characterization of iPSC-derived RPE. A) RPE cells were derived from a commercial control iPSC line (Control RPE-1) and two independent clonal iPSC lines from patient without retinal disease (Control RPE-2 and -3). RPE monolayers were fixed 6 weeks after plating. Micrographs show positive immunostaining immunostaining for the RPE markers MerTK, RPE65, Bestrophin, ZO1, $Na^{+}/K+$ ATPase, CRALBP, MITF and tyrosinase in RPE monolayers derived from all three control lines (red and green signals). Nuclei were stained with DAPI (blue signal). Upper and lower panels in the first three columns show the green and red channels from the same field of double immunostained RPE cells. The fourth column panels show two separately stained RPE samples from each line. **B**) RPE cells were derived from three independent clonal iPSC lines derived from the proband patient. RPE monolayers were fixed for immunostaining 6 weeks after plating. Micrographs show positive immunostaining for the RPE markers MerTK, RPE65, Bestrophin, ZO1, $Na^{+}/K+$ ATPase, CRALBP, MITF and tyrosinase in RPE monolayers derived from all three patient lines (red and green signals). Nuclei were stained with DAPI (blue signal). Upper and lower panels in the first three columns show the green and red channels from the same field of double immunostained RPE cells. The fourth column panels show two separately stained RPE samples from each line. **C**) Expression levels of the RPE markers *RPE65*, *MERTK*, *MITF*, *BEST1* and *PAX6* were measured in RPE cells derived from the three control (dark grey bars) and three patient (light grey bars) iPSC lines by qRT-PCR. RPE derived from an adult 37 year old female donor was used as a positive control (black bars). **D**) Representative micrographs showing immunostaining for primary cilia in control and patient RPE. RPE monolayers were stained with ARL13B (red signal), pericentrin (green signal) and DAPI (blue signal).
