# Supplementary material for: Gene replacement therapy restores RCBTB1 expression and cilium length in patient‐derived retinal pigment epithelium
Source: J Cell Mol Med. 2021 Oct 7;25(21):10020–7. doi: 10.1111/jcmm.16911 (PMC8572767; doi:10.1111/jcmm.16911)
Supplement: Supplementary file 2 — Table S1 [file JCMM-25-10020-s001.pdf]

Suplementary Table 1: Primer sequences

**Target**	**Forward/Reverse Primer Sequences**
RPE65	TTTGGCACCTGTGCTTTCCCAG/GTTGGTCTCTGTGCAAGCGTAG
MERTK	AGACTTCAGCCACCCAAATG/GGGCAATATCCACCATGAAC
MITF	GGCTTGATGGATCCTGCTTTGC/GAAGGTTGGCTGGACAGGAGTT
BEST1	TGCCAACCTGTCAATGAAGGCG/TCCAGTCGTAGGCATACAGGTG
PAX6	AACGATAACATACCAAGCGTGT/GGTCTGCCCGTTCAACATC
RCBTB1	AGCCAGTGAAGCACTGTACG/TTGGTCGTCCCATTCCCAAG
CUL3	TGTGGAGAACGTCTACAATTTGG/GCGCCTCTGTCTACGACTT
NFE2L2	GTTGCCCACATTCCCAAATC/CGTAGCCGAAGAAACCTCAT
RXRA	GACGGAGCTTGTGTCCAAGAT/AGTCAGGGTTAAAGAGGACGAT
IDH1	AGAAGCATAATGTTGGCGTCA/CGTATGGTGCCATTTGGTGATT
SLC25A25	AGAATGATGGACGCATTGAC/ATGGTCATCGTGCCGTTT
GAPDH	AGAAGGCTGGGGCTCATTTG/AGGGGCCATCCACAGTCTTC
