# Supplementary material for: Gene replacement therapy restores RCBTB1 expression and cilium length in patient‐derived retinal pigment epithelium
Source: J Cell Mol Med. 2021 Oct 7;25(21):10020–7. doi: 10.1111/jcmm.16911 (PMC8572767; doi:10.1111/jcmm.16911)
Supplement: Supplementary file 3 — Table S2 [file JCMM-25-10020-s002.pdf]

Supplementary Table 2: Antibodies used

	Antibody	Dilution	Company Cat # and RRID
RPE markers	Mouse anti-RPE65	1/200	Abcam Cat# ab13826, RRID:AB_2181006561766
	Rabbit anti-BEST1	1/200	Abcam Cat# ab14928, RRID: AB_301519
	Mouse anti-ZO-1	1/100	Invitrogen Cat#339194, RRID: AB_2533147
	Rabbit anti-MERTK	1/100	Abcam Cat# ab52968, RRID: AB_2143584
	Mouse anti-MITF	1/200	Invitrogen Cat#MA5-14154, RRID: AB_10982126
	Mouse anti-CRALBP	1/200	Abcam Cat# ab15051, RRID: AB_2269474
	Rabbit anti-Na^+/K^+-ATPase	1/100	Cell signalling #3010, RRID: AB_2060983
	Mouse anti-Tryosinase	1/100	Abcam Cat#738,RRID: AB_305899
Cilia markers	Rabbit anti-Arl13B	1/200	Proteintech Cat#17711-1-AP, RRID: AB_2060867
	Mouse anti-Pericentrin	1/200	Abcam Cat#ab28144, RRID:AB_2160664
Secondary antibodies	Alexa Fluor 488 Goat anti-mouse	1/500	Thermo Fisher Scientific, Cat# A28175, RRID AB_2536161
	Alexa Fluor 546 Goat anti-rabbit	1/500	Molecular Probes Cat# A-11035, RRID:AB_143051
	Alexa Fluor 488 Goat anti-rabbit	1/500	Molecular Probes Cat# A-11008, RRID: AB_143165
	Alexa Fluor 546 Goat anti-mouse	1/500	Molecular Probes Cat# A-11003, RRID: AB_141370
